# Sepsis Inflammation Impairs the Generation of Functional Dendritic Cells by Targeting Their Progenitors

**DOI:** 10.3389/fimmu.2021.732612

**Published:** 2021-09-09

**Authors:** Jie Lu, Kun Sun, Huiping Yang, Dan Fan, He Huang, Yi Hong, Shuiyan Wu, HuiTing Zhou, Fang Fang, YanHong Li, Lijun Meng, Jie Huang, Zhenjiang Bai

**Affiliations:** ^1^Department of Pediatric Intensive Care Unit, Children Hospital of Soochow University, Suzhou, China; ^2^Department of Emergency, Xuzhou Children’s Hospital, Xuzhou Medical University, Xuzhou, China; ^3^Department of Pediatrics, Changshu Hospital Affiliated to Nanjing University of Chinese Medicine, Suzhou, China; ^4^Institute of Pediatric Research, Children’s Hospital of Soochow University, Suzhou, China; ^5^Department of Nephrology, Children’s Hospital of Soochow University, Suzhou, China; ^6^Department of Cardiovascular Medicine, Children Hospital of Soochow University, Suzhou, China

**Keywords:** sepsis, dendritic cells, dendritic cell progenitors, common dendritic cell progenitors, G-CSF, IFN-γ

## Abstract

**Background:**

Sepsis is a complex systemic immune dysfunction syndrome induced by infection. Sepsis has a high mortality rate, with most patients dying due to systemic organ failure or secondary infection. Dendritic cells (DCs) are professional antigen-presenting cells. Upon infection with microbes, DCs are activated to induce adaptive immune responses for controlling infection. DC generation and function are impaired during sepsis; however, the underlying mechanisms remain largely unknown.

**Methods:**

Peripheral blood samples from sepsis patients were collected to examine DC subsets, DC progenitors, and apoptosis of DCs by flow cytometer. *In vitro* induction of DCs from hematopoietic stem/progenitor cells were established and a variety of sepsis-associated inflammatory mediators [e.g., interferon-gamma (IFN-γ), interleukin-1beta (IL-1β), tumor necrosis factor-alpha (TNF-α) and granulocyte-colony stimulating factor (G-CSF)] and Lipopolysaccharide (LPS) were determined for the impact on DC generation and function *in vitro*.

**Results:**

Our results demonstrate that sepsis-induced systemic inflammation impairs the capacity of hematopoietic stem and progenitor cells (HSPCs) to produce DCs, including conventional DCs (cDCs) and plasmacytoid DCs (pDCs). We investigated peripheral blood (PB) samples from 34 pediatric patients on days 1 to 7 following diagnosis. Compared to healthy donors (n = 18), the sepsis patients exhibited a significantly fewer percentage and number of pDCs and cDCs, and a lower expression of antigen presenting molecule HLD-DR and co-stimulatory molecules (e.g., CD86) on the surface of DCs. This sepsis-induced DC impairment was associated with significantly increased apoptotic death of DCs and marked decreases of progenitor cells that give rise to DCs. Furthermore, we observed that among the tested sepsis-associated cytokines (e.g., IFN-γ, IL-1β, TNF-α, and G-CSF), G-CSF and IFN-γ impaired DC development from cultured HSPCs. G-CSF also markedly decreased the expression of HLA-DR on HSPC-derived DCs and their cytokine production, including IL-12 and IFN-β.

**Conclusions:**

Collectively, these findings indicate that sepsis impairs the survival of functional DCs and their development from HSPCs. Strategies for improving DC reconstitution following sepsis may restore DC progenitors and their associated function.

## Introduction

Sepsis is a form of life-threatening organ dysfunction due to a dysregulated host immune response to infection (1). In 2017, an estimated 48.9 million cases of sepsis were recorded worldwide, with 11.0 million sepsis-related patient deaths, representing 19.7% of all global deaths (2). Dendritic cells (DCs) are the most potent antigen presentation cells (APCs), which play an essential role in the pathogen recognition, regulation of immune response, and inflammation (3, 4), and link both the innate and adaptive immunity (5, 6). DCs are mainly classified as conventional dendritic cells (cDCs) and plasmacytoid dendritic cells (pDCs) in peripheral blood (7). DC defects and dysfunction represent an important contributor to persistent inflammation, immunosuppression, susceptibility to infection and death in sepsis patients (8). An autopsy analysis has shown that adult sepsis patients have fewer DCs in the blood and spleen compared to non-sepsis patients (6, 9, 10). In addition, sepsis patients with low DC counts are susceptible to nosocomial infections (11), suggesting the DC compartment may play an important role during sepsis progression. However, there is limited clinical data regarding DC generation and function in the acute inflammatory phase in children with sepsis. A more in-depth understanding of the mechanisms by which DC generation and function are impaired during and after sepsis will be important for improving the outcomes of sepsis therapy.

Sepsis induces a systemic dysregulated inflammatory response that is characterized by the excessive production of inflammatory mediators [e.g., interleukin-1β (IL-1β), interferon-γ (IFN-γ), tumor necrosis factor-α (TNF-α), granulocyte-Colony stimulating factor (G-CSF), interleukin-10 (IL-10) and transforming growth factor-β (TGF-β)] and the inflammatory inducer [lipopolysaccharide (LPS)] (12). Accumulating evidence indicates that inflammatory factors cause DC impairment, dysfunction, and apoptosis (13–22). DCs develop from hematopoietic stem/progenitor cells (HSPCs) in the human bone marrow (BM) through successive lineage commitment and differentiation steps: multipotent progenitors (MPPs); common myeloid progenitors (CMPs); granulocyte macrophage DC progenitors (GMDPs); monocyte and DC progenitors (MDPs); and common DC progenitors (CDPs) (23, 24). Under a pathological microenvironment, DC development in the bone marrow (BM) may have been dramatically changed in response to inflammatory stimuli (25). The level of the pro-inflammatory factors G-CSF and IFN-γ, are low under steady state conditions, but were elevated in response to inflammatory stimuli (26–29). Moreover, elevated levels of G-CSF and IFN-γ have been reported to be associated with a poor clinical outcome in sepsis (30–34). Several studies have shown that inflammation may inhibit the regenerative capacity of HSCs and DC progenitor cells (35–37). However, whether the *de novo* generation of DCs from HSPCs is impaired in sepsis within this complex internal environment remains largely unknown.

In the present study, we examined the DC survival capability and DC progenitors in the peripheral blood (PB) of sepsis patients. Sepsis severely impairs the generation of CDPs and depletes DCs in the PB of pediatric patients. We found that among sepsis-associated inflammatory cytokines (e.g., IL-1β, IFN-γ, TNF-α and G-CSF) and LPS, G-CSF and IFN-γ were found to significantly reduce DC development and functional differentiation. These findings identify a previously uncharacterized mechanism by which sepsis impairs DC generation and function. Strategies to improve DC reconstitution following sepsis may be required to restore DC progenitors and their function.

## Methods

### Healthy Donors and Patients

The present study was conducted in the pediatric intensive care unit (ICU) of the Children’s Hospital of Soochow University. A total of 34 critically ill patients with sepsis were enrolled from January 2020 to April 2021. The sepsis patients’ demographic is shown in **Table 1**. The sepsis patients were further divided into two groups based on the time following diagnosis: early stage (days 1−2, n = 27) and later (days 3−7, n = 13). This study was approved by the Medical Ethics Committee of the Children’s Hospital of Soochow University (Suzhou, China). Written informed consent was obtained from children with sepsis (or their parents) upon their initial admission to the hospital and from healthy volunteers.

**Table 1:** Characteristics of the 34 children with sepsis included in the study.

	Sepsis (n = 34)
Age (years)	1.04 [0.20-3.62]
Male gender [n (%)]	22 (64)
Site of initial infection [n (%)]	
Blood stream	7 (20.6)
Lung	2 (5.9)
Abdomen	11 (32.4)
Brain	10 (41.7)
Multi-site	2 (5.9)
Unidentified infection	2 (5.9)
Principal diagnosis besides sepsis	
Encephalitis	15 (44.1)
Gastroenteritis	5 (14.7)
MODS	4 (11.8)
Urinary Tract Infection	3 (8.8)
Cellulitis	2 (5.9)
Hemolytic anemia	1 (2.9)
Choledochal cysts	1 (2.9)
Perianal abscess	1 (2.9)
Renal abscess	1 (2.9)
Polyarteritis nodosa	1 (2.9)
Prism-III score	9 [3-14.25]
ICU-free days in 30 days	5 [3-10.25]
Mortality [n (%)]	4 (11.8)

Values are expressed as median [interquartile range], or a number (percentage)

MODS, Multiple Organ Dysfunction syndrome; PRISM-III, pediatric risk of mortality score-III.

### Inclusion and Exclusion Criteria

Pediatric patients with sepsis were included in this study if they met the diagnostic criteria for sepsis (1, 38). Patients were excluded from the study if they had the following diseases: congenital immunodeficiency disease; immunodeficiency caused by human immunodeficiency virus (HIV) infection; BM or solid organ transplantation; hematologic malignancy; and allergic diseases (e.g., asthma).

### Cell Isolation and Flow Cytometry

Fresh leukocytes were isolated from the peripheral blood (PB) after using red blood cell lysis buffer (Solarbio, Beijing, China). Samples were incubated with fluorescence-labeled antibodies for the directed analysis on the Attune NxT Flow Cytometer (Life Technologies, CA, USA). All mAbs used for fluorescence staining were purchased from Biolegend (San Diego, CA) or Invitrogen (Carlsbad, CA) (**Table 2**). mAb staining was performed as previously described (39). Total DCs were characterized as negative for lineage markers (CD3, CD14, CD15, CD16, CD19, and CD56) and positive for HLA-DR. Among these cells, CD1c^+^ cells were defined as conventional DCs (CD1c^+^ cDCs), whereas CD123^+^CD1c^-^CD11c^-^ cells were defined as plasmacytoid dendritic cells (pDCs) (7). HSPCs were labelled as CD34^+^ cells. Human common DC progenitors (CDPs) were identified as CD34^+^CD38^-^CD10^-^CD45RA^+^CD123^+^CD115^-^. Human monocyte-DC progenitors (MDPs) were characterized as CD34^+^CD38^-^CD10^-^CD45RA^+^CD123^int^CD115^-^. Human granulocyte-monocyte DC progenitors (GMDPs) were marked as CD34 ^+^CD38^-^CD10^-^CD45RA^+^CD123^int^CD115^+^ (40).

**Table 2:** Antibodies.

Marker	Fluorochrome	Clone	Manufacturer	Cat.no.	Isotype
HLA-DR	APC/CY7	L243	Biolegend	307618	Mouse IgG2a
CD3	FITC	SK7	Biolegend	344804	Mouse IgG1
CD14	FITC	HCD14	Biolegend	325604	Mouse IgG1
CD15	FITC	HI98	Biolegend	301904	Mouse IgM
CD19	FITC	HIB19	Biolegend	302206	Mouse IgG1
CD20	FITC	2H7	Biolegend	302304	Mouse IgG2b
CD56(NCAM)	FITC	MEM-188	Biolegend	304604	Mouse IgG2a
CD86	PE/CY7	II2.2	Biolegend	305422	Mouse IgG2b
CD123	APC/CY7	6H6	Biolegend	306012	Mouse IgG1
CD1C	PE/CY7	L161	Biolegend	331506	Mouse IgG1
CD11C	Pacific Blue	Bu15	Biolegend	337212	Mouse IgG1
CD14	Pacific Blue	HCD14	Biolegend	325616	Mouse IgG1
CD115(CSF-1R)	APC	9-4D2-1E4	Biolegend	347323	Rat IgG1
CD10	FITC	HI10a	Biolegend	312207	Mouse IgG1
CD38	PE	HB-7	Biolegend	356603	Mouse IgG1
CD34	APC	561	Biolegend	343607	Mouse IgG2a
CD45RA	Pacific Blue	HI100	Biolegend	304129	Mouse IgG2b
CD123	PE/CY7	6H6	Biolegend	306010	Mouse IgG1

### Detection of Apoptosis

The evaluation of apoptotic cells was examined using FITC-conjugated *Annexin-V* and propidium iodide (PI) kits (Invitrogen, Carlsbad, CA). Living (*Annexin*-*V* ^−^ *PI* ^−^), early apoptotic (*Annexin*-*V* ^+^ *PI* ^−^) and late apoptotic or necrotic (*Annexin*-*V* ^+^ *PI* ^+^) cells were distinguished.

### DC Induction and Generation From HSPCs

All recombinant cytokines were purchased from PeproTech (PeproTech, NJ). HSPCs were purified from G-CSF mobilized human PB using CD34^+^ microbeads (Miltenyi, 130046702) in accordance with the manufacturer’s instructions. To induce DCs, HSPCs were first cultured in Roswell Park Memorial Institute (RPMI) 1640 containing 10% fetal bovine serum (Dongling Biotech), FMS-like tyrosine kinase 3 ligand (FLT3L) (100 ng/mL), stem cell factor (SCF) (20 ng/mL), interleukin-3 (IL-3) (20 ng/mL), and thermoplastic polyolefin (TPO) (20 ng/mL) for 7 days, followed by additional culture for 7 days following the removal of TPO. On day 14, the cells were collected for analysis. To test the impact of inflammatory mediators [e.g., IFN-γ (10ng/ml), IL-1β (10ng/ml), G-CSF (10ng/ml) and TNF-α (10ng/ml)] and LPS (10ng/ml) on DC development, we cultured HSPCs as described above with or without addition of the inflammatory cytokines and the inducer as described above. To induce DC activation, we added LPS (100 ng/mL, Sigma), R848 (100 ng/mL, Invitrogen) or CpG oligonucleotide (CPG ODN) (1 μM) into the DC population or fluorescence-activated cell sorting (FACS)-sorted purified DC population using a BD Influx or BD FACs Aria II.

### Real-Time RT- PCR

Total RNA was extracted from DCs derived from HSPCs *in vitro* using Trizol (Invitrogen) according to the manufacturer’s instructions. Reverse-transcription was performed using a >commercial kit with random primers (Takara). Complementary DNA (cDNA) was quantified through quantitative real-time polymerase chain reaction (PCR) using a SYBR Green PCR mix (Takara) on a LightCycler 480 PCR System (Roche). The thermocycler conditions included an initial hold period at 95°C for 10 min, followed by a three-step PCR program, as follows: 95°C for 20s, 55°C for 30s, and 72°C for 30s for 40 cycles. Transcript abundance was calculated using the delta Ct method (normalization with 18S). All of the primer sequences are listed in **Table 3**.

**Table 3:** Primer for real-time RT-PCR.

Gene name	Primer sequence	
18s	Forward	5′-GCTGCTGGCACCAGACTT-3′
	Reverse	5′-CGGCTACCACATCCAAGG-3′
IL12	Forward	5′-CCAGCACATTGAAGACCTGT-3′
	Reverse	5′-CAGGGTCATCATCAAAGACG-3′
Irf4	Forward	5′-CCACAGAGCCAAGCATAAGG-3′
	Reverse	5′-CCGGTAGTACAGGCAGATGT-3′
Ifna	Forward	5′-TCATTTCTCCTGCCTGAAGG-3′
	Reverse	5′-GAGGACAGAGATGGCTTGAG-3′
Ifnb	Forward	5′-TTGACATCCCTGAGGAGATTAAGC-3′
	Reverse	5′-TTAGCCAGGAGGTTCTCAACAATAG-3′
Irf8	Forward	5′-AGGGGACAAAGCTGAACCAG-3′
	Reverse	5′-CAGTTGCCACGCCTAGTTTG-3′
Tcf4	Forward	5′-CAAATAGAGGAAGCGGGGCA-3′
	Reverse	5′-CTGTGCCTGCTGAGAGAGAT-3′
Batf3	Forward	5′-GGATGATGACAGGAAGGTCCG-3′
	Reverse	5′-GTGTTTTCTTGCTCCAGGCTC-3′
Flt3	Forward	5′-TGCCGCTGCTCGTTGTTTT-3′
	Reverse	5′-GAGGTCTTCCGGGGATTCTG-3′
Zebf46	Forward	5′-TCCCTGCTGTTCGAGTACCT-3′
	Reverse	5′-GCATGTGTCGCTTGAGGATG-3′
Cebpa	Forward	5′-GGACCCTCAGCCTTGTTTGT-3′
	Reverse	5′-AGACGCGCACATTCACATTG-3′

### Statistics

Statistical analysis was performed using Graph Pad Prism 8 software (San Diego). Continuous data were expressed as the mean ± standard deviation (S.D.). Data conforming to a normal distribution were compared using a two-tailed t test, whereas non-normally distributed data were evaluated using a Mann-Whitney U-test. A threshold of *p* < 0.05 was considered to be statistically significant.

## Results

### Sepsis Induces a Selective Reduction of DCs in Pediatric Patients

**Figure 1:**
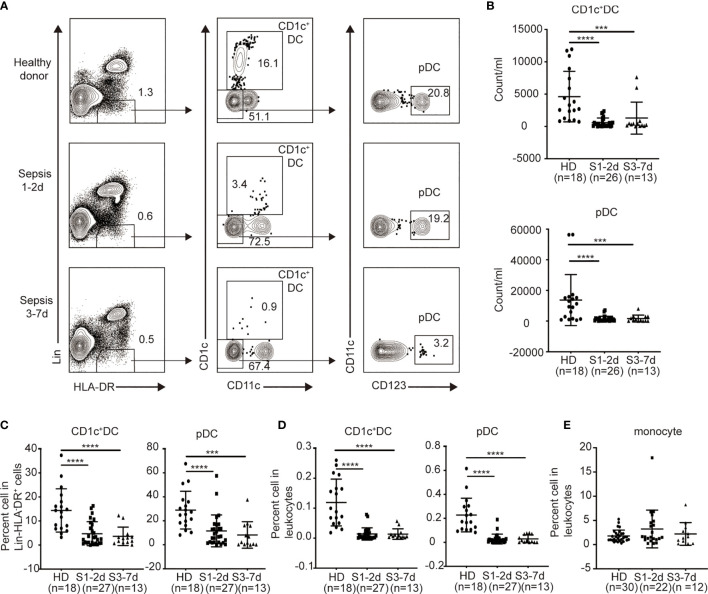
The percentage and number of circulating blood DCs from sepsis patients are selectively declined. Leukocytes from healthy donors and sepsis patients were obtained and directly stained for DC surface markers. **(A)** Gating strategy for the identification of human DC subsets from healthy donors (n = 18), sepsis 1-2d (n = 27) and sepsis 3-7d (n = 13). **(B)** The absolute number of each DC subset in per ml peripheral blood among the three groups. **(C)** Percentage of each DC subset among Lin^-^HLA-DR^+^ cell population among the three groups. **(D)** Percentage of each DC subset in leukocytes among the three groups. **(E)** Percentage of monocytes in leukocytes among the three groups: healthy donors (n = 30), sepsis 1-2d (n = 22) and sepsis 3-7d (n = 12). Error bars indicate mean ± SD. ***P < 0.001; ****P < 0.0001.

To identify the effect of sepsis on DCs, we obtained PB from sepsis patients (n = 34) (**Table 1**). We focused on CD1c^+^ DCs (CD1c^+^CD123^-^CD11c^+^) and pDCs (CD1c^-^CD123^+^CD11c^-^) (**Figure 1A**), which represent the majority of the DCs found in the PB (7). PB from normal healthy donors (n = 18) was assessed as a control. Both the percentage and number of CD1c^+^ DCs and pDCs significantly declined in sepsis patients during the first two days following diagnosis compared to that of the healthy donors (**Figures 1B−D**). There was approximately an 8- to 10-fold reduction in both the frequency and number of CD1c^+^DCs and pDCs out of the total leukocytes of sepsis patients compared to that of the healthy donors (**Figures 1B, D**). Notably, this DC-associated defect in the sepsis patients persisted throughout 7 days after disease onset (**Figures 1B−D**). Sepsis did not significantly alter the frequency of monocytes in the PB throughout 7 days when DCs were decreased (**Figure 1E**). These data indicate that sepsis causes severe DC defects in the PB early after disease onset and does not recover within 7 days.

### Sepsis Down-Regulates CD86 and HLA-DR on the Surface of PB DCs

**Figure 2:**
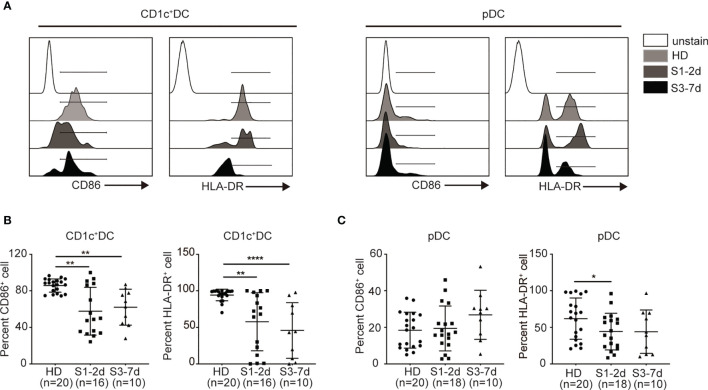
Sepsis down-regulates the expression of CD86 and HLA-DR on DCs in PB. **(A)** Flow cytometry histograms of CD86 and HLA-DR expression on each DC subset. **(B, C)** Percentage of CD86^+^ and HLA-DR^+^ cells on each DC subset from healthy donors (n = 20), sepsis 1-2d (n = 16) and sepsis 3-7d (n = 10). Error bars indicate mean ± SD. *P < 0.05; **P < 0.01; ****P < 0.0001.

The expression of antigen-presenting molecules (e.g., HLA-DR) and co-stimulatory molecules (e.g., CD86) on DCs is important to T cell priming in response to infection (41, 42). To this end, we examined the surface expression HLA-DR and CD86 on DCs derived from the PB of sepsis patients (**Figure 2A**). CD1c^+^ DCs from sepsis patients expressed lower levels of CD86 and HLA-DR during early stage of the disease and remained at significantly lower levels at the later stage compared to their counterparts in the healthy donors (**Figure 2B**). The expression of HLA-DR on the surface of pDCs derived from the children with sepsis in the early stage of disease was significantly lower compared with that of the health donor group, whereas the level of CD86 did not change significantly (**Figure 2C**). Therefore, sepsis also impairs DC maturation and activation, affecting CD1c^+^ cDCs to a greater extent. There are very few dendritic cells in human peripheral blood, and some cells will not survive overnight after stimulant, it is really difficult to detect the cytokine release function of dendritic cells on a technical level.

### Sepsis Induces DC Apoptosis

**Figure 3:**
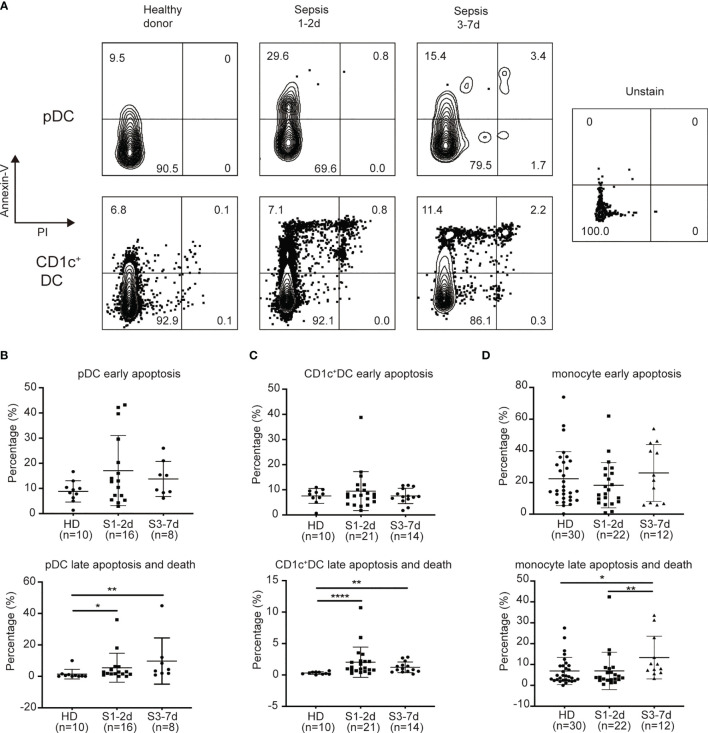
Sepsis patients exhibit increased levels of late apoptosis on DC subsets rather than on monocytes. **(A)** Flow detection of apoptosis of DC subsets. **(B)** Percentage of early apoptotic pDC and late apoptotic/dead pDC from healthy donors(n = 10), sepsis 1-2d (n = 16) and sepsis 3-7d (n = 8). **(C)** Percentage of early apoptotic CD1c^+^cDC and late apoptotic/dead CD1c^+^cDC from healthy donors (n = 10), sepsis 1-2d (n = 21) and sepsis 3-7d (n = 14). **(D)** Percentage of early apoptotic monocytes and late apoptotic/dead monocytes from healthy donors (n = 30), sepsis 1-2d (n = 22) and sepsis 3-7d (n = 12). Error bars indicate mean ± SD. *P < 0.05; **P < 0.01; ****P < 0.0001.

Previous studies have demonstrated that lymphocyte apoptosis is associated with immune deficiency in sepsis (43, 44). Both death-receptor- and mitochondrial-mediated pathways have been found to be responsible for sepsis-induced apoptosis, suggesting the engagement of multiple cell death stimuli (45). We hypothesized that sepsis-induced DC defects may be the result of increased rates of cell apoptosis. To address this possibility, we obtained PB from sepsis patients to examine DC apoptosis, and used healthy donors as controls. Flow cytometric analysis of Annexin-V and PI staining revealed live cells (*Annexin*-*V* ^−^ *PI* ^−^), early apoptotic cells (*Annexin*-*V* ^+^ *PI* ^−^), late apoptotic cells (*Annexin-V* ^+^ *PI* ^+^), and necrotic cells (*Annexin*-*V* ^−^ *PI* ^+^) (**Figure 3A**). We found that approximately 2% of CD1c^+^DCs and 5.5% of pDCs from sepsis patients at days 1 to 3 of diagnosis were late apoptotic cells, whereas healthy donors had approximately 6-fold and 3-fold fewer late apoptotic cells among the CD1c^+^ DC (0.3%) and pDC (1.4%) subsets, respectively (**Figure 3C**). From days 3 to 7 of sepsis, the frequency of late apoptotic cells among the CD1c^+^ DCs and pDCs was approximately 4-fold and 7-fold greater than that of healthy donors (**Figures 3B, C**). During the early apoptotic stage, we no significant difference in CD1c^+^ DCs and pDCs was observed between healthy donors and sepsis patients. In contrast, sepsis did not markedly affect the survival capability of monocytes (**Figure 3D**). These findings suggest that sepsis increases the rate of DC apoptotic cell death throughout the acute inflammation phase.

### CDPs Dramatically Decrease During Sepsis

**Figure 4:**
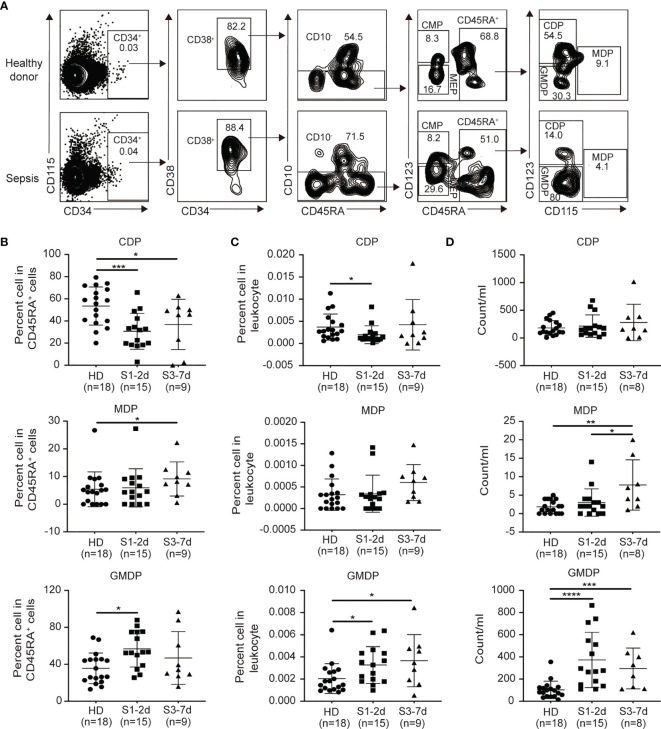
Decreases in CDPs and increases in GMDPs from sepsis patients are observed. **(A)** Flow cytometry of human peripheral blood, showing the gating strategy of GMDPs, MDPs, CDPs. **(B, C)** The proportion of CDPs, MDPs and GMDPs in CD45RA^+^ cells and total leukocytes respectively from healthy donors (n = 18), sepsis 1-2d (n = 15) and sepsis 3-7d (n = 9). **(D)**The absolute number of CDPs, MDPs and GMDPs in per ml PB from healthy donors(n = 18), sepsis 1-2d (n = 15) and sepsis 3-7d (n = 8). Error bars indicate mean ± SD. *P < 0.05; ***P < 0.001; ****P < 0.0001.

DCs develop from HSPCs in the BM. Under the steady-state conditions, HSCs give rise to multipotent progenitors (MPPs), which can become granulocyte macrophage DC progenitors (GMDPs), monocyte and DC progenitors (MDPs), and common DC progenitors (CDPs) (40). Of these, CDPs directly differentiate into both CD1c^+^DCs and pDCs (46–48). Therefore, we next asked whether the impaired generation of DC progenitors may contribute to DC defects in the PB during sepsis. Three major DC progenitors (i.e., GMDPs, MDPs and CDPs) were observed in the PB from sepsis patients and healthy donors (**Figure 4A**). Sepsis patients exhibited a significant decrease in the frequency of CDPs between days 1 to 7 post-diagnosis compared to the healthy donors (**Figures 4B−D**). In contrast, the sepsis patients showed an increased frequency and number of MDPs and GMDPs was potentially enhanced *in vivo* (**Figure 4C**). Thus, CDPs are more sensitive than GMDPs and MDPs to sepsis-mediated suppression.

### Generation of Human CD1c^+^ cDCs and pDCs From Human HSPCs in Culture

**Figure 5:**
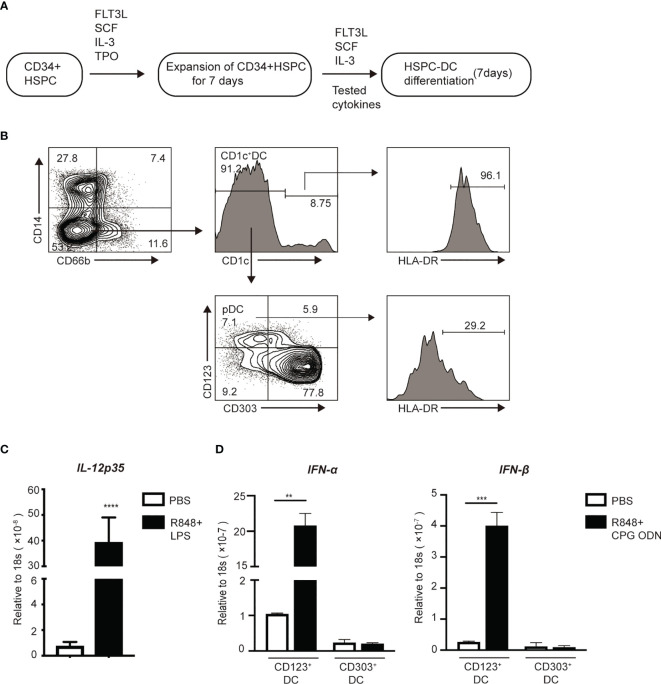
*In vitro* induction of DCs from HSPCs. **(A)** Workflow for the induction of HSPC-DCs. **(B)** Flow cytometry of pDCs (CD66b^-^CD14^-^CD1c^-^CD123^+^CD303^-^) and CD1c^+^cDCs (CD66b^-^CD14^-^CD1c^+^) populations after 14 days of induction from CD34^+^HSPCs. On day 14, **(C)** After stimulated with the TLR4 and TLR7 agonists for 2 hours, mRNA of FACS sorter purified CD1c^+^cDCs was extracted to measure gene expression using real time RT-PCR. **(D)** CD123^+^pDCs and CD303^+^pDCs were highly purified using FACs sorter. Then both populations were stimulated with the TLR7 and TLR9 for 2 hours, mRNA was collected to measure gene expression using real time RT-PCR. Error bars indicate mean ± SD. **P < 0.01; ***P < 0.001; ****P < 0.0001.

To better understand the mechanisms by which inflammatory stimuli impact the generation of DC progenitors from HSPCs, we established an *ex vivo* culture method of producing DCs from human CD34^+^ HSPCs (**Figure 5A**) (40). On day 14 of culture, the cells were collected and stained for antibodies to identify DCs and DC subsets. CD66b and CD14 were used to exclude granulocytes and monocytes, respectively. CD1c^+^ DCs were CD66b^-^CD14^-^ HLA-DR^+^ CD1c^+^ (**Figure 5B**). Upon stimulation with the TLR4 agonist, LPS, these cells significantly upregulated 80-fold more IL-12p35 mRNA (**Figure 5C**), suggesting the induction of CD1c^+^ DCs. pDCs are specialized cells that produce of high levels of IFN-α and IFN-β (49). We identified pDCs based on their CD66b^-^CD14^-^CD1c^-^CD123^+^CD303^+^ phenotype, sorted them into CD123^+^ and CD303^+^ cell subsets respectively, and stimulated them with R848 and CPG ODN for 2 h. mRNA was extracted from these pDCs to examine the expression of genes encoding IFN-α and IFN-β. Upon CpG stimulation, we observed that CD123^+^ pDCs rather than CD303^+^ cells, could rapidly upregulate the expression of mRNA encoding IFN-α and IFN-β (**Figure 5D**). Therefore, CD66b^-^CD14^-^ HLA-DR^+^CD1c^+^ cDCs and CD66b^-^CD14^-^CD1c^-^CD123^+^ pDCs were used for all subsequent studies.

### G-CSF Impairs the Generation of CD1c^+^cDCs and pDCs From HSPCs *In Vitro*

**Figure 6:**
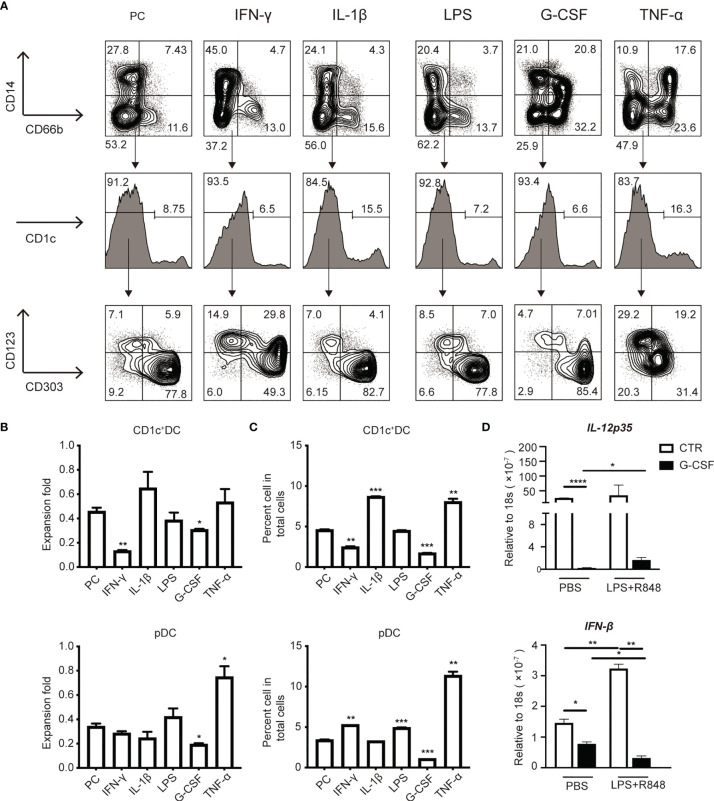
G-CSF and IFN-γ impaired the induction of HSPC-DCs subsets. **(A)** Flow cytometry of HSPC-DCs subset from various tested cytokines [e.g., IFN-γ (10ng/ml), IL-1β (10ng/ml), G-CSF (10ng/ml) and TNF-α (10ng/ml)] and LPS (10ng/ml), which were added into the culture system on day7. **(B)** The graphs showed the proliferation of HSPC-pDCs and CD1c^+^cDCs under various cytokines stimulation by day14. **(C)** The percentage of HSPC-pDCs and CD1c^+^ cDCs among total populations under various cytokine stimulation on day14. **(D)** Total HSPC-DCs were stimulated with the TLR4 and TLR7 agonists for 2 hours, mRNA was extracted to measure gene expression using real time RT-PCR. Data shown are representative of three independent experiments. Error bars indicate mean ± SD. *P < 0.05; **P < 0.01; ***P < 0.001; ****P < 0.0001.

To identify the potential factors that impair DC generation in sepsis patients, we screened a panel of inflammatory cytokines known to play an important role in sepsis for their ability to affect DC induction in *in vitro* culture (**Figure 6A**). As shown in **Figures 5B, C**, the addition of LPS and IL-1β, which are known to be important factors that contribute to sepsis (28, 50), did not significantly affect DC generation from HSPCs (**Figures 6B, C**). G-CSF, which is also increased during acute inflammation (27, 28, 51), significantly reduced the number and frequency of CD1c^+^ DCs and pDCs in culture (**Figures 6B, C**). IFN-γ is a crucial pro-inflammatory cytokine that is produced in response to pathogen infection (52). Our results revealed that IFN-γ inhibited total DC proliferation, which primarily affected CD1c^+^ DCs. In light of the impact of IFN-γ on the differentiation of DC subsets, we were surprised to find that IFN-γ had different effects on CD1c^+^ DCs and pDCs, suppressing CD1c^+^ DCs but promoting pDC differentiation (**Figures 6B, C**). Notably, TNF-α profoundly increased pDC generation in culture (**Figures 6B, C**). Collectively, these data suggest that both G-CSF and IFN-γ have the capacity to impair DC generation from cultured HSPCs. Furthermore, IFN-γ inhibits CD1c^+^ DCs, but promotes pDC differentiation. Moreover, increased IL-12p35 and IFN-β production following stimulation with LPS/R848 further supports the observed impaired generation of authentic CD1c^+^ DCs and pDCs. (**Figure 6D**).

### G-CSF Impairs the Expression of Pro-DC Transcription Factors in Cultured Human HSPCs *In Vitro*

**Figure 7:**
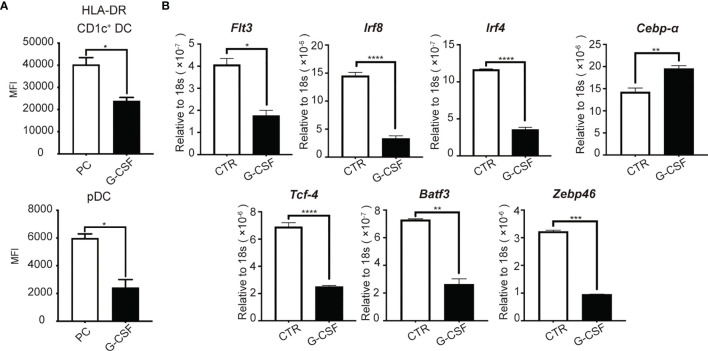
G-CSF impairs the expression of pro-DC development transcription factors in cultured human HSPCs *in vitro*. On D14 of culture, **(A)** HLA-DR expression on HSPC-DC subsets after G-CSF treatment for 7 days. **(B)** mRNA was collected to measure gene expression using real time RT-PCR. Results are representative of three independent experiments. Error bars indicate mean ± SD. *P < 0.05; **P < 0.01; ***P < 0.001; ****P < 0.0001.

We next examined whether G-CSF may affect the function of DCs generated in culture. It has been previously reported that G-CSF levels are associated with a poor clinical outcome in patients with sepsis (30–32). We found that unstimulated DCs generated in the presence of G-CSF expressed lower levels of HLA-DR compared to their counterparts produced in the absence of G-CSF (**Figure 7A**). This finding suggests that G-CSF may contribute to decreased HLA-DR expression on DCs in sepsis patients.

To examine the mechanisms associated with G-CSF-mediated suppression of DC generation from HSPCs, we examined the impact of G-CSF on the expression of transcription factors, including *IRF8, IRF4, TCF4, BATF3*, and *ZBTB46*. TCF4 and IRF4 are known to promote pDC development (53). In addition, Batf3 is highly important for cDC1 development (54), IRF4 determines CD1c^+^ cDC2 differentiation (55), and ZBTB46 has been identified as a marker of both cDC1 and cDC2 (56, 57). We assessed the level of transcription factor and Flt3 expression by real-time RT-PCR. FLT3 and the transcriptional factor, IRF8, are known to be important throughout the entire process of DC development (58–60). We found that G-CSF significantly reduced the expression of FLT3 and IRF8 (**Figure 7B**), which was consistent with the reduction of proliferation and differentiation of CD1c^+^ DCs and CD123^+^ pDCs. We also observed a marked decrease in the expression of the genes associated with cDCs (*ZBTB46, IRF4*, and *BATF3*) and pDCs (*TCF4* and *IRF4*) (**Figure 7B**). *CEBPA*, which functions to promote myeloid differentiation (61, 62), was also increased (**Figure 7B**). These results indicate that G-CSF impairs pDC and CD1c^+^ cDC generation from HSPCs, likely through regulation of the key transcription factors required for DC development.

## Discussion

In this study, we demonstrated that sepsis induces DC defects in pediatric patients. These defects are associated with the impaired generation of CDPs and increased apoptosis of DCs. Among the tested inflammation-associated cytokines, we found that both G-CSF and IFN-γ contribute to reduced DC generation from HSPCs in culture. Moreover, the addition of G-CSF significantly decreases the expression of transcription factors required for HSPC-derived DC production. Given the crucial role of DCs in both innate and adaptive immunity, our findings are important for gaining a better understanding of the pathology associated with sepsis-associated immune suppression in patients.

A lower number of DCs in sepsis appear to be associated with poor clinical outcomes. Guisset et al. and Grimaldi et al. have shown that sepsis patients with depleted circulating DCs are more likely to develop septic shock, and can even predict higher mortality rates in fetal sepsis (9, 11). Our study found that increased DC apoptosis and death can in part explain the depletion of DCs. During inflammation, the local microenvironment, especially inflammatory cytokines, is important for the regulation of DC function and survival (63, 64). The study by Raffray et al. demonstrated that the serum from patients with sepsis induced higher DC death compared to serum from normal healthy donors or patients with cardiogenic shock (65). Furthermore, accumulating evidence has shown that inflammatory factors (e.g., G-CSF, IFN-γ, TNF-α, IL-6, IL-10, and TGF-β) can cause DC impairment and dysfunction, even inducing the apoptosis of DCs (13–22). Our results showed that DC apoptosis occurred during sepsis.

In addition to apoptosis, we also provide evidence that sepsis may have a significant impact on the differentiation of DC progenitors into DCs. This represents another mechanism by which sepsis causes a reduction of DCs. Our findings are supported by observations in mouse models. For example, Pasquevich et al. and Beshara et al. reported that BM CDPs were reduced in mice infected with Ye/Ec/Sa/Lm (*Yersinia enterocolitica/Escherichia coli/Staphylococcus aureus/Listeria monocytogenes*) (66) and influenza A virus (IVA), respectively (67). Conversely, the study by Macal et al. showed that CDPs appeared to increase on day 5, but were subsequently reduced in mice during infection with chronic lymphocytic choriomeningitis virus (68). This disparity may be due to different timings of examination and infection models. Collectively, our studies and these of others indicate that sepsis may have a significant impact on hematopoiesis and DC development in the BM. To our knowledge, our study is the first to report that DC progenitors are decreased in the PB of sepsis patients. Our results further reveal a possible underlying cause of this defect may be due to enhanced myelopoiesis (e.g., monocytes and granulocytes), increased circulating GMDPs and MDP production. Several studies have reported an increase in neutrophils and monocytes during sepsis (69–71). This primarily results from ‘emergency hematopoiesis’ characterized by converting the hematopoietic response program to replenish depleted granulocytes and monocytes following a systemic infection (25).

Although we have explored the reduction of DCs associated with increased apoptosis and impaired generation of DC progenitors, we cannot rule out the possibility that decreased DCs in the PB may be attributable to the altered migration capacity of DCs, which cannot be investigated in humans.

Our findings reveal that sepsis impairs the number and antigen-presentation functionality of DCs, leading to a reduction in immature DCs, which may further perpetuate immunosuppression. DCs display an immature phenotype under steady state conditions. Following stimulation with microbes or danger signals, DCs undergo activation and maturation through a series of phenotypic and functional changes, including the upregulation of the expression of surface MHC-II and co-stimulatory molecule CD86 (72, 73). We found that the downregulation of these molecules suggests a defect in the antigen-presenting capacity of DCs, which is to a large extent influenced by the microenvironment as previously discussed (13–22). These tolerogenic immature DCs impair the ability of the immune system to mount a T cell response against secondary infections (74–76). Some studies have demonstrated that tolerogenic DCs are decreased in sepsis, which are susceptible to secondary infection, even if critically ill patients recover from systemic inflammatory response syndrome (SIRS) (11, 77). Thus, the downregulation of HLA-DR and CD86 are suggestive of a state of immune suppression in sepsis (78). Consistent with our observations, the studies by Grimaldi et al. (11) and Poehlmann et al. (6) showed that cDCs exhibit lower expression of HLA-DR, whereas there was no difference in its expression on pDCs in patients with sepsis. This difference may be due to the low expression of MHC-II and co-stimulatory molecules on pDCs (79). It is likely that CD1c^+^ DCs are more sensitive than pDCs for predicting the severity of sepsis. Future studies will use a larger cohort of patients to strengthen our conclusion.

We also report for the first time, that G-CSF markedly suppresses the generation and functionality of CD1c^+^ DCs and pDCs. Recent reports show that G-CSF not only mobilizes HSCs from the BM into the peripheral blood, but also modifies the bone marrow microenvironment (80, 81). In addition to the observation that G-CSF facilitates the proliferation, differentiation, and maturation of neutrophils to treat or prevent neutropenia in clinical patients (82–85), our results demonstrated that G-CSF inhibited the differentiation and proliferation of CD1c^+^ DCs and pDCs. In contrast to our results, Shaughnessy et al. showed that the administration of G-CSF in eight patients with graft-versus-host disease (GVHD) could mobilize more pDCs into the PB (86). The discrepancy may result from DC development modulated by the complex *in vivo* environment, whereas our research focuses on the effect of the signaling of G-CSF on DC development. We also found that G-CSF could impair the activation and maturation of DCs by HLA-DR, as well as the cytokine secretion function of DCs (e.g., IL-12, and IFN-β). Similar to our results, Reddy et al. reported that the administration of G-CSF to mice resulted in donor DCs that produced low levels of TNF-α and IL-12 in acute GVHD (22). Another study reported that G-CSF-stimulated PBMCs of healthy donors contained predominantly T helper 2-inducing DCs (19, 87). These results suggest that G-CSF may alter DC functionality. The impact of G-CSF on HSC differentiation into DCs has not been previously examined in-depth. Our results demonstrate that G-CSF inhibited the differentiation and development of CD1c^+^ DCs and pDCs. These results prompted us to investigate DC depletion when sepsis patients were administrated G-CSF as a treatment for neutropenia.

Furthermore, our results demonstrate that G-CSF is associated with key transcriptional factors required for DC development. Our results showed that both *FLT3* and *IRF8* expression regulating the DC development were decreased. Similarly, the expression of genes that regulate cDCs (*ZBTB46, BATF3*, and *IRF4*) and pDCs (*TCF4* and *IRF4*), respectively, were also declined. However, the gene, *CEBPA*, which promotes granulopoiesis, was increased. These findings suggest that G-CSF may play a dual role, promoting granulocyte lineage differentiation (71, 88, 89), as well as inhibiting DC development and functionality. Additional studies are needed to elucidate the mechanism by which G-CSF regulates these DC-related transcriptional factors.

Our results indicate that the pro-inflammatory factor, IFN-γ, also plays an important role in the differentiation and development of DCs. Previous studies have reported that IFN-γ has a pro-inflammatory potential in sepsis (90, 91) and can even drive immune suppression and induce secondary infections (33). Moreover, many studies have shown that IFN-γ directly regulates the differentiation and function of HSCs during infection, but not during steady-state hematopoiesis (37, 92, 93). For example, during acute lymphocytic choriomeningitis virus (LVMV) infection, IFN-γ impairs HSC self-renewal and restoration of the number of HSCs (93). During a chronic infection, IFN-γ activates quiescent HSCs (37) and induces myeloid differentiation to defend against infection (92, 94). In addition, it has been reported that IFN-γ may upregulate the expression of MHC-class I/II on DCs and promote the production of cytokines important for T cell-mediated immune responses. Indeed, a lack of IFN-γ signaling in mature IFNR^-^/^-^ DCs results in the reduced expression of intercellualr cell adhesion molecule-1 (ICAM-1), CD86, IL-1β, and IL-12p70 (95, 96). In addition to its role in DC activation during the acute phase of sepsis, IFN-γ can exert tolerogenic effects in DCs during the later phase (18, 97). There are currently a limited number of reports that have investigated the effects of IFN-γ on the generation of DC subsets. Consistent with our study, Laustsen et al. found that IFN-γ priming promoted HSPC generation of pDCs (98). Interestingly, we also found that IFN-γ mainly suppresses CD1c^+^ DC proliferation and differentiation. To our knowledge, this is the first report describing this effect of IFN-γ on CD1c^+^ DC generation.

In conclusion, our study demonstrates that the impaired generation of CDPs and increased apoptosis rates of DCs contribute to the DC defects observed in sepsis patients. It is likely that the production of G-CSF and IFN-γ during sepsis plays an important role in suppressing DC development and differentiation. These findings may have important implications for improving the understanding of both the pathology and immunosuppression associated with sepsis.

## Data Availability Statement

The raw data supporting the conclusions of this article will be made available by the authors, without undue reservation.

## Ethics Statement

The studies involving human participants were reviewed and approved by Medical Ethics Committee of the Children’s Hospital of Soochow University. Written informed consent to participate in this study was provided by the participants’ legal guardian/next of kin.

## Author Contributions

JL, KS, LM, JH, and ZB conceived and designed the project. JL, KS, HY, DF, HH, and HY performed experiments. JL, KS, HZ, FF, YL, SW, LM, JH, and ZB analyzed and interpreted the data. JL, LM, JH, and ZB wrote the manuscript. JL, KS, HZ, FF, YL, SW, LM, JH, and ZB edited the manuscript. All authors contributed to the article and approved the submitted version.

## Funding

The work was supported by National Natural Science Foundation (Grant number 81971867), Science and Technology Project of Jiangsu Province (BE2019672), the Natural Science Foundation of Jiangsu Province (Grant BK20190053), the Medical Science Program of Jiangsu Province (Grant H2019002), the Science and Technology Program of Suzhou (Grant SYS2018067) and Health Talent Training Program of Suzhou (GSWS2020044).

## Conflict of Interest

The authors declare that the research was conducted in the absence of any commercial or financial relationships that could be construed as a potential conflict of interest.

## Publisher’s Note

All claims expressed in this article are solely those of the authors and do not necessarily represent those of their affiliated organizations, or those of the publisher, the editors and the reviewers. Any product that may be evaluated in this article, or claim that may be made by its manufacturer, is not guaranteed or endorsed by the publisher.

## Abbreviations

DC, dendritic cell; HSPC, hematopoietic stem and progenitor cell; cDC, conventional DC; pDC, plasmacytoid DC; PB, peripheral blood; HLA-DR, human leukocyte antigen DR; APC, antigen presentation cell; MPP, multipotent progenitor; CMP, common myeloid progenitor; GMDP, granulocyte macrophage DC progenitor; MDP, monocyte and DC progenitor; CDP, common DC progenitor; Ye, Yersinia enterocolitica; Ec, Escherichia coli; Sa, Staphylococcus aureus; Lm, Listeria monocytogenes; IVA, influenza A virus; HIV, human immunodeficiency virus; LVMV, Lymphocytic choriomeningitis virus; GVHD, Graft-versus-host disease; SIRS, Systemic inflammatory response syndrome; MODS, Multiple Organ Dysfunction syndrome; PRISM-III, pediatric risk of mortality score-III; IL-1β, interleukin-1β; IFN-γ, interferon-γ; TNF-α, tumor necrosis factor-α; G-CSF, granulocyte-Colony stimulating factor; LPS, lipopolysaccharide; IL-10, interleukin-10; TGF-β, transforming growth factor-β; FLT3L, FMS-like tyrosine kinase 3 ligand; SCF, stem cell factor; IL-3, interleukin-3; TPO, thermoplastic polyolefin; PD-L1, programmed cell death-Ligand 1; IDO, indoleamine 2,3-dioxugenase.

## Glossary

**Table:**

DC	dendritic cell
HSPC	hematopoietic stem and progenitor cell
cDC	conventional DC
pDC	plasmacytoid DC
PB	peripheral blood
HLA-DR	human leukocyte antigen DR
APC	antigen presentation cell
MPP	multipotent progenitor
CMP	common myeloid progenitor
GMDP	granulocyte macrophage DC progenitor
MDP	monocyte and DC progenitor
CDP	common DC progenitor
Ye	Yersinia enterocolitica
Ec	Escherichia coli
Sa	Staphylococcus aureus
Lm	Listeria monocytogenes
IVA	influenza A virus
HIV	human immunodeficiency virus
LVMV	Lymphocytic choriomeningitis virus
GVHD	Graft-versus-host disease
SIRS	Systemic inflammatory response syndrome
MODS	Multiple Organ Dysfunction syndrome
PRISM-III	pediatric risk of mortality score-III
IL-1β	interleukin-1β
IFN-γ	interferon-γ
TNF-α	tumor necrosis factor-α
G-CSF	granulocyte-Colony stimulating factor
LPS	lipopolysaccharide
IL-10	interleukin-10
TGF-β	transforming growth factor-β
FLT3L	FMS-like tyrosine kinase 3 ligand
SCF	stem cell factor; IL-3: interleukin-3
TPO	thermoplastic polyolefin
